# Changes in Stress-Strain Index in School-Aged Children: A 3-Year Longitudinal Study

**DOI:** 10.1155/2023/6680748

**Published:** 2023-10-14

**Authors:** Zhengfei Yang, Bo Wu, Zhouyue Li, Mengting Yu, Jinyun Jiang, Shuyuan Chen, Shengsong Xu, Junwen Zeng, Mengyi Wang, Xiao Yang

**Affiliations:** ^1^State Key Laboratory of Ophthalmology, Zhongshan Ophthalmic Center, Sun Yat-sen University, Guangzhou, China; ^2^South China Hospital, Shenzhen University, Shenzhen, China

## Abstract

**Purpose:**

To determine three-year change of the corneal biomechanical parameter stress-strain index (SSI) in schoolchildren aged 7– 9 years and their correlation with refractive error and axial length (AL).

**Methods:**

This is a prospective cohort study. Data of the AL, refractive error, and corneal biomechanical parameter SSI were collected at baseline and a 3-year follow-up for 217 schoolchildren. SSI, AL, and refractive error were measured via corneal visualization Scheimpflug technology (Corvis ST), IOLMaster biometry, and cycloplegic refraction. Three years of changes in SSI and its association with refractive error and AL were analyzed. Participants were divided into persistent nonmyopia (PNM), newly developed myopia (NDM), and persistent myopia (PM). The three-year difference in SSI among the three groups was analyzed.

**Results:**

After three years of follow-up, the corneal biomechanical parameter SSI decreased in all participants (*P* < 0.01). There was a negative correlation between the change in SSI and the change in AL (*r* = −0.205, *P*=0.002) and a positive correlation between the change in refractive error (*r* = 0.183, *P*=0.007). After three years of follow-up, there was a decrease in the SSI for the NDM, PM, and PNM participants, with a median change of −0.05 for PNM and −0.13 and −0.09 for the NDM and PM, respectively. There was a significant decrease in corneal biomechanical properties for NDM patients compared with PNM patients (*P* < 0.01).

**Conclusion:**

In 7- to 9-year-old schoolchildren, SSI decreased after three years of the longitudinal study, and the change in SSI was correlated with the change in AL and refractive error. There was a rapid decrease in corneal biomechanical properties among newly developed myopic patients.

## 1. Introduction

Myopia is a global public health problem and its progression can lead to visual impairment, such as myopic macular degeneration and retinal detachment. Previous studies have shown that progressive myopia reduced the elasticity, stress, and load-bearing capacity of the sclera [1, 2]. Human scleral biomechanics is measured mainly in vitro, which can only reflect the state of myopia after stabilization but not during development [1]. Nguyen et al. studied human and donor eyes and found that both corneal and scleral material properties contribute to the observed corneal response to air-puff-induced deformation [3]. It has been hypothesized that the biomechanical properties of the cornea may reflect those of the sclera since the corneal and the sclera tissue of the eye are structurally connected [4].

Prior studies have demonstrated that the biomechanical characteristics of the cornea were influenced by both central corneal thickness (CCT) and intraocular pressure (IOP) [5–7]. It is therefore difficult to obtain accurate corneal biomechanical measurements that are unaffected by CCT and IOP [8]. Eliasy et al. developed a novel biomechanical parameter, known as the material stiffness parameter stress-strain index (SSI), for the estimation of the in vivo elastic properties of the cornea [8]. There exists a significant correlation between the SSI value and the stiffness of the cornea. Specifically, when the SSI value increases, the corneal stiffness also tends to increase, and conversely, when the SSI value decreases, the corneal stiffness tends to decrease [8]. SSI is also a marker for material stiffness and corneal biomechanical parameters, which do not correlate with the CCT or IOP [8].

Recent cross-sectional studies found that eyes with higher myopia have lower corneal stiffness [9, 10] and may be less stable and more susceptible to stress [11–13]. Similarly, in a cross-sectional study conducted by Liu et al., it was observed that the SSI value exhibited a sequential decrease when categorized according to the degree of myopia, namely, low, moderate, high, and ultra-high [14]. The difference in the rate of change of spherical equivalent (SE) refractive error may be related to the change in corneal stiffness and needs to be investigated in a longitudinal study. Results have been mixed from a limited number of longitudinal studies in this area. Shah et al. found no significant association between changes in corneal biomechanical properties and axial length (AL) or SE over two years [15]. In contrast, Momeni-Moghaddam et al. reported a decrease in most of the corneal biomechanical parameters after a four-year follow-up [16]. It is noteworthy that they utilized the ocular response analyzer, which could not differentiate corneal behavior from the effects of IOP and CCT [17]. A longitudinal study is warranted to investigate the causality relationship between corneal biomechanics and axial length using corneal biomechanical parameters that are independent of IOP and CCT.

Therefore, the current study that included participants aged 7–9 years was designed to explore the changes in SSI in a three-year longitudinal cohort and investigate the potential role of the SSI in the development of myopia.

## 2. Methods

### 2.1. Study Design

Data for this study were collected as part of an ongoing prospective study that was approved by the institutional review board of Sun Yat-sen University School of Public Health. The study was conducted in accordance with the Declaration of Helsinki. Parents were informed about the study's purpose and content before obtaining their written consent.

In November and December 2018, 12 primary schools in Guangzhou City, China, randomly selected Grade 2 and Grade 3 students to participate in the study. After three years of follow-up, the study was completed in November and December 2022.

This study excluded participants with strabismus, amblyopia, and nystagmus. Participants who used orthokeratology were also excluded. This study measured AL using a laser interferometer (IOLMaster 500; Zeiss). Cycloplegic autorefraction was performed by an autorefractor (KR 8800; Topcon Corp.). A cyclopentolate solution of 1% was administered three times over five minutes to induce cycloplegia. Following complete cycloplegia, measurements of the refractive index were taken. We also performed cycloplegic subjective refraction on children with vision worse than 20/20 in either eye.

A child with persistent nonmyopia (PNM) was defined as having a SE value greater than −0.50D at both visits. Newly developed myopia (NDM) was defined as a child who was nonmyopic (SE > −0.50D) at baseline but had myopia (SE ≤ −0.50D) at the 3-year follow-up visit. A child who was myopic (SE ≤ −0.50D) at both visits was defined as having persistent myopia (PM) [18].

### 2.2. Corvis ST and SSI

A visual dynamic intraocular pressure analyzer, Corvis ST (software version 1.6b2507), was used to measure corneal biomechanical properties. A quality score of “OK” was used for the analysis of all measurements. A repeat examination was conducted if the quality was not rated “OK.” Subjects whose multiple attempts did not meet an “OK” assessment were excluded.

Software used least squares analysis and numerical modeling to estimate SSI. Parameters such as CCT, bIOP, and SP-HC were used as inputs [8].

The equation of SSI reported in the Eliasy et al.'s study was SSI = *f*(*a*_1_ + *a*_2_*C*_1_ + *a*_3_*C*_2_ + *a*_4_*C* + *a*_5_*C*_1_*C'*_2_ + *a*_6_*C*+ *a*_7_*C* + *a*_8_*CC*_2_ + *a*_9_*C*_1_*C* + *C*) + ln (SP-HC)) [8]. The C1 and C2 are obtained by dividing the values of CCT and bIOP by 545 and 20, respectively. The natural logarithm of the difference between SP and HC, denoted as Ln (SP-HC), is calculated. The constants a1 to a9 are determined through a fitting process, where the equation is adjusted to match the given numerical input and output values [8].

### 2.3. Data Analysis

Statistics analysis was performed by IBM SPSS (IBM SPSS Statistics version 25.0, IBM SPSS, USA). The Shapiro–Wilk test was performed to check data normality. Right eyes were the only ones included for Spearman analysis since the right and left eyes showed high correlations (r, 0.91 for SE and 0.88 for AL). We analyzed baseline and follow-up differences using Kruskal–Wallis H tests. The Wilcoxon test was used to compare the difference between baseline and follow-up. Spearman correlation was used to analyze the correlation among SSI, refraction error, and AL. A *P* value lower than 0.01 was considered statistically significant.

## 3. Results

### 3.1. General Characteristics

217 schoolchildren with a median age of 8.1 years at baseline, of which 52.07% were girls, were available for analysis. Boys had a greater AL than girls at baseline and the follow-up visit (*P* < 0.001). No significant differences were observed in SSI and SE between genders (Table 1).

After a three-year follow-up period, the SSI decreased significantly (*P* < 0.001), the eye elongated significantly, and myopia shifted significantly (Table 1). There was no significant difference between the 7.0–8.0 age groups and the 8.1–9.0 age groups in these ocular parameters (Table 2).

### 3.2. Relationship between Changes in the SSI and Other Ocular Components

There was a positive correlation between changes in the SSI and SE (*r* = 0.183, *P*=0.007) and a negative correlation between changes in the SSI and AL (*r* = −0.205, *P*=0.002) (Table 3).

### 3.3. SSI Changes over Time among Refractive Status Groups

The 217 participants were divided into 92 PNMs, 97 NDMs, and 28 PMs. At baseline, PMs showed lower SSI values than PNMs; NDMs and PNMs showed no significant differences (Table 4). After three years of follow-up, all participants decreased in SSI, with PNMs losing 0.05, NDM 0.13, and PMs 0.09; the decrease of SSI in NDM was greater than in PNM. SE and AL changed more significantly in the NDM and PM than in the PNM (Table 4).

## 4. Discussion

This study found that after three years of follow-up, the corneal biomechanical parameter stress-strain index decreased as the degree of myopia increased. The change in SSI correlated with the change in SE and AL. Moreover, there was no significant difference between the PNM and NDM groups in the baseline. After three years of follow-up, however, a significant difference was observed between the PNM and NDM groups, suggesting that an increase in axial length may lead to a decrease in corneal biomechanical properties. In addition, patients with newly developed myopia showed greater attenuation in corneal stiffness than those with persistent nonmyopia.

Our study found that SSI decreased after three years of follow-up. This decrease can be attributed to the significant impact of axial elongation. In addition, the change in SSI was negatively correlated with the change in axial length, which is typically associated with myopia development. Momeni-Moghaddam et al. also found a similar difference with the ocular response analyzer (ORA) [16]. However, a two-year change in AL/SE and CH/CRF was not significantly associated with Shah et al.'s study [15]. The difference between our study and Shahe et al.'s study may be because the shift of AL and SE in our study was more prominent (0.82 mm and −2.0 DS) than in theirs (0.22 mm and −0.7 DS).

One of the major findings from this three-year longitudinal study was that the change of SSI in newly developed myopia is larger than in persistent nonmyopia. The dramatic decrease of the SSI in the NDM indicates that the corneal biomechanical properties also underwent dramatic changes with myopia increase.

Many cross-sectional studies have found that the SSI value was lower in eyes with higher spherical error [9, 10, 19]. In our longitudinal study, SSI in the NDM group showed lower values than PNM after three years of follow-up, indicating that SSI changes are secondary to the increase in axial length. The findings suggest that as children progress from emmetropia to myopia, there is a discernible decrease in SSI. However, it remains to be explored whether SSI can reliably indicate children at risk of developing myopia. NDM-related factors, such as reduced outdoor activity and heightened educational pressure, as well as excessive smartphone use, have been identified as influencing myopia development [20]. Notably, greater outdoor activity appears to be associated with a reduced risk of myopia [20]. In addition, higher degrees of myopic refractive error and longer axial lengths correlate with increased screen time on smartphones and computers [21]. These lifestyle factors likely contribute to the emergence of NDM. While this study did not incorporate underlying lifestyle factors that differentiate between PNM and NDM, investigating the causal relationships between myopia, lifestyle, and corneal biomechanics promises to advance our understanding of myopia progression. Future research endeavors should further explore the intricate interplay of lifestyle factors, myopia progression, and corneal biomechanics. The changes in corneal collagenous may be physiological factors of corneal biomechanical decrease in newly developed myopia. Scleras are fibrous collagenous shells that are continuous with the cornea anteriorly, creating essentially closed chambers in the anterior, equatorial, and posterior eye. Scleral thinning occurs very rapidly in response to the onset of myopia development [2]. Thus, corneal biomechanics—inseparable from whole-eye biomechanics—may undergo a decrease in corneal biomechanical properties.

Age-associated biomechanical changes in the cornea have been reported in a few studies in vivo. In our study, there was no difference in the biomechanical parameters of SSI in relation to age, similar to Matalia et al.'s study, where corneal deformation parameters were unaffected by age in vivo [22]. However, glycation-induced cross-linking of collagen molecules has been shown in other studies to cause corneal stiffening as people age [23, 24]. According to Kamiya et al.'s study, corneal biomechanical parameters decline significantly with age [25]. Matalia et al.'s study also showed that corneal biomechanical parameters stiffer with increasing age [22, 26]. Discrepancies in patient ages may explain this discrepancy in the results between us. In Kamiya et al. and Elsheikh et al.'s studies, the participants were with a wider range, aged 19–89 years old and 50–95 years old, respectively. In our study, a narrower age (7–9 years) of children was included; the two age groups in our study may be too close to produce a statistical difference. Furthermore, a study revealed that SSI exhibited a notable level of stability before age 35, followed by an increase as individuals increased in age [27]. The alteration in corneal biomechanics observed with advancing age may be attributed to modifications in the molecular composition of the cornea, which show an increased trend of collagen glycation and collagen molecules in individuals aged ≥40 years [23, 24, 28].

There is a recognized limitation in our study. We only included participants of Grade 2 and Grade 3, with a limited age and refraction range. Further research should be conducted involving a wider range of ages and refractions to gain a better understanding of the topic.

In summary, by conducting a study with a 3-year follow-up, we observed the role of the corneal biomechanical parameter stress-strain index during myopia development in schoolchildren and found that stress-strain index decreases and is associated with an increase in myopia. Moreover, a rapid attenuation of SSI was observed in newly developed myopic patients, suggesting its potential significance in myopia onset. Future research should consider including different age groups to provide a more comprehensive understanding. In addition, investigating the intricate relationship among lifestyle factors, myopia progression, and corneal biomechanics warrants further exploration in our upcoming studies.

## Figures and Tables

**Table 1:** The characteristics of the 217 participants at baseline and during the 3-year follow-up.

Parameters	Total	Boys	Girls	*Z*	*P* value
	*n* = 217	*n* = 104	*n* = 113		
*SSI*					
Baseline	1 (0.91, 1.1)	0.98 (0.89, 1.08)	1.02 (0.94, 1.12)	−1.91	0.06
Follow-up	0.92 (0.83, 1.01)	0.93 (0.83, 1.01)	0.91 (0.84, 1.01)	−0.04	0.97
Change	−0.09 (−0.18, 0.01)^*∗*^	−0.07 (−0.16, 0.03)^*∗*^	−0.11 (−0.19, 0)^*∗*^	−1.89	0.06

*SE*					
Baseline	0.88 (0.38, 1.25)	0.88 (0.38, 1.25)	0.88 (0.5, 1.25)	−0.10	0.92
Follow-up	−0.75 (−2.25, 0.13)	−0.56 (−2.01, 0.25)	−1 (−2.25, 0)	−1.48	0.14
Change	−1.63 (−2.5, −1)^*∗*^	−1.38 (−2.38, −0.75)^*∗*^	−1.88 (−2.63, −1.25)^*∗*^	−2.63	0.01

*AL*					
Baseline	22.95 (22.49, 23.51)	23.29 (22.84, 23.8)	22.65 (22.37, 23.04)	−6.04	<0.001^#^
Follow-up	23.82 (23.3, 24.51)	24.07 (23.52, 24.77)	23.59 (23.16, 24.2)	−3.49	<0.001^#^
Change	0.82 (0.57, 1.18)^*∗*^	0.76 (0.46, 1.14)^*∗*^	0.95 (0.65, 1.24)^*∗*^	−2.72	0.01

*Age*					
Baseline	8.1 (7.82, 8.39)	8.11 (7.81, 8.37)	8.09 (7.88, 8.41)	−0.52	0.60
Follow-up	11.02 (10.74, 11.31)	11.03 (10.74, 11.29)	11 (10.76, 11.32)	−0.44	0.66
Change	2.92 (2.92, 2.94)^*∗*^	2.93 (2.92, 2.94)^*∗*^	2.92 (2.92, 2.94)^*∗*^	−0.51	0.61

AL, axial length; SE, spherical equivalent; SSI, stress-strain index. ^#^*P* < 0.01, *P* value for comparison between boys and girls using a Mann–Whitney *U* test. ^*∗*^*P* < 0.01 for comparison between baseline and follow-up using a Wilcoxon test.

**Table 2:** The baseline and follow-up characteristics of the 217 participants.

Parameters	7.0–8.0 years old	8.1–9.0 years old	*Z*	*P* value
	*n* = 89	*n* = 128		
*AL*				
Baseline	22.84 (22.41, 23.41)	23 (22.54, 23.52)	−1.312	0.189
Follow-up	23.71 (23.16, 24.59)	23.86 (23.34, 24.5)	−0.614	0.539
Change	0.82 (0.61, 1.24)	0.8 (0.52, 1.16)	−1.259	0.208

*SSI*				
Baseline	0.98 (0.89, 1.08)	1.01 (0.92, 1.11)	−1.252	0.210
Follow-up	0.94 (0.84, 1.01)	0.92 (0.83, 1.01)	−0.504	0.615
Change	−0.08 (−0.17, 0.04)	−0.09 (−0.19, −0.01)	−1.090	0.276

*SE*				
Baseline	0.75 (0.13, 1.13)	1 (0.5, 1.28)	−2.303	0.021
Follow-up	−0.88 (−2.63, 0)	−0.75 (−1.91, 0.25)	−1.467	0.142
Change	−1.63 (−2.63, −0.88)	−1.63 (−2.25, −1)	−0.527	0.598

AL, axial length; SE, spherical equivalent; SSI, stress-strain index. *P* < 0.01, *P* value for comparison between boys and girls using a Wilcoxon test.

**Table 3:** Correlation coefficients between changes in ocular components and baseline characteristics (*r* value).

	SSI, baseline	SSI, change	SE, baseline	SE, change	AL, baseline	AL, change	Age, baseline
SSI, change	−0.621^*∗∗*^						
SE, baseline	0.112	0.162^*∗∗*^					
SE, change	0.018	0.183^*∗∗*^	0.387^*∗∗*^				
AL, baseline	−0.107	0.036	−0.409^*∗∗*^	−0.058			
AL, change	−0.01	−0.205^*∗∗*^	−0.508^*∗∗*^	−0.833^*∗∗*^	0.139^*∗*^		
Age, baseline	0.065	−0.062	0.126	0.082	0.093	−0.111	
Age, change	0.226^*∗∗*^	−0.036	0.361^*∗∗*^	0.179^*∗∗*^	−0.095	−0.234^*∗∗*^	0.011

AL, axial length; SE, spherical equivalent; SSI, stress-strain index; ^*∗*^correlation is significant at the 0.01 level (2-tailed).

**Table 4:** Changes in stress-strain index among the refractive groups.

			PNM (*n* = 92)	NDM (*n* = 97)	PM (*n* = 28)	^*∗*^ *P* value
SSI	Baseline	Median (range)	1.02 (0.93, 1.12)	1.01 (0.91, 1.09)	0.95 (0.86, 1.06)	0.047
	Follow-up	Median (range)	0.98 (0.89, 1.06)	0.90 (0.81, 0.98)	0.85 (0.81, 0.91)	<0.001
	Change	Median (range)	−0.05 (−0.14, 0.03)^#^	−0.13 (−0.22, −0.01)^#^	−0.09 (−0.14, −0.06)^#^	0.016

AL (mm)	Baseline	Median (range)	22.72 (22.47, 23.14)	22.97 (22.43, 23.52)	23.78 (23.09, 24.18)	<0.001
	Follow-up	Median (range)	23.37 (22.96, 23.71)	24.13 (23.59, 24.75)	24.85 (24.57, 25.51)	<0.001
	Change	Median (range)	0.51 (0.43, 0.65)^#^	1.15 (0.9, 1.35)^#^	1.16 (1.02, 1.48)^#^	<0.001

SE (D)	Baseline	Median (range)	1.25 (1, 1.5)	0.63 (0.5, 1)	−1.19 (−2, −0.63)	<0.001
	Follow-up	Median (range)	0.25 (0, 0.75)	−1.5 (−2.5, −0.88)	−3.38 (−4.59, −3)	<0.001
	Change	Median (range)	−0.94 (−1.25, −0.63)^#^	−2.25 (−2.88, −1.75)^#^	−2.38 (−2.63, −2.19)^#^	<0.001

AL, axial length; SE, spherical equivalent; SSI, stress-strain index; NDM, newly developed myopia; PM, persistent myopia; PNM, persistent nonmyopia. ^*∗*^Statistical significance was tested using Kruskal–Wallis H. ^#^*P* < 0.01, compared between baseline and follow-up within the group tested using a Wilcoxon test.

## Data Availability

The data used to support the findings of this study are available from the corresponding author upon request.
